# Supplementary material for: Chromothripsis during telomere crisis is independent of NHEJ, and consistent with a replicative origin
Source: Genome Res. 2019 May;29(5):737–49. doi: 10.1101/gr.240705.118 (PMC6499312; doi:10.1101/gr.240705.118)
Supplement: Supplemental Material [file supp_gr.240705.118_Supplemental_file_1.zip › contigs/annotated_contigs/DB84/contig.2.DB84_length_674_mean_cov_7.78931750742.docx]

**DB84_length_674_mean_cov_7.78931750742**

```
GTCAAAGCTGGGCTGTTCTTGTGTCATGAAACTCACTGAAGCAGCTGAGTTATTATATTGGTGAAATTAATTATTAAAATGAAGACATG
 >chr8:36334392-36334834 + E=3e-254
CTTTGTTCTGCTTTAAAAGAAAGATAAAAGCTTGAACAATTAAAAATGGTTTTCTATTATAGCTAATGTGCAATATGGATTCCAACATA

ATTAGTTAAGTCTGCAAAATTATTTTTATATTATCTAAGGCTTCATTTAACACATTTATACATTCATTCATTTATTCGCTCAAAAATAT

TGTCTTCTGAGCACCAAATCTTGTGAAGGAAAATGGAAATATAAAAGTAAGTAAAACTTAGGTCTTGTTGTTTGTAAGCTCTTTACTTA

ATATATCTATGAATTCCCTTGGTCCAGGAAGTAAACAGGGTGTTAAATGGACCGTCTGTGGTTCTCTTTCCAAAAACCTGACGTT|T|A
                                                                                      >
TAATAATAAAATAATAATAATAATTGAGTGACTCCCACAGGAACAATGAGTACCTCTAACTGACCCGATCTCTGTTTCAAGATACTAAT
chr8:36338930-36339163 + E=2e-125
TTTATCTATAATCGTCTAGGCTTCCTGGAGAAATAAGTGATTCCAGGTAAAACTTCCAGGCAAGCTTTGGACAGTTTAGTGTGCTGGAA

AGCAAAGATCTATTTAAAGCATAATTAAGTTGTGTCAATAGAAGTTAGGATCT
```
